# Mycorrhizal Abundance and Its Interaction with Cereal Root Traits and Crop Productivity in Organically Managed Cereal/Legume Intercropping

**DOI:** 10.3390/plants14233561

**Published:** 2025-11-21

**Authors:** Agnė Veršulienė, Andrius Garbaras, Gražina Kadžienė, Arman Shamshitov, Monika Toleikienė

**Affiliations:** 1 Institute of Agriculture, Lithuanian Research Centre for Agriculture and Forestry (LAMMC), Instituto al. 1, Kėdainiai District, LT-58344 Akademija, Lithuania; arman.shamshitov@lammc.lt (A.S.); monika.toleikiene@lammc.lt (M.T.); 2 Isotopic Research Laboratory, Department of Nuclear Research, Center for Physical Sciences and Technology, Savanorių 231, LT-02300 Vilnius, Lithuania; andrius.garbaras@ftmc.lt

**Keywords:** spring barley, oat, physical root parameters, arbuscular mycorrhizal fungi, crop productivity

## Abstract

Mixed cropping may positively affect soil fertility and soil biological activities, such as those related to mycorrhizal colonization intensity (M%), which plays a vital role in the plant nutrient cycle and can improve tolerance to drought and pathogens. This plant and soil fungi symbiosis helps to reduce dependency on chemical fertilizers, promotes sustainable agricultural practices, and minimizes environmental impacts. However, field studies that clearly assess the effects of cereal/legume intercropping on mycorrhizal intensity and relate it to plant productivity, yield quality, and plant adaptation to climate change are lacking. This field experiment was conducted to assess the effects of cereals/legume intercropping on mycorrhizal colonization, and to explore its interaction with physical cereal root parameters and crop yield. Three main crops, spring barley, oat, and field pea, were grown as monocultures. For the spring barley and oat, the study also included two different fertilization levels (with and without organic fertilizers) and legume intercropping (field pea and red clover). The intercropping had a significant impact on spring barley and oat root length, diameter, and specific root length. The general average of root length and diameter was higher in oat–pea and barley–pea cropping systems. The most significant effect in root architecture parameters observed in red clover was when it was intercropped with barley or oat. The establishment of field pea intercrop significantly increased M% in spring barley and had a positive effect on the grain yields of both spring barley and oat. Meanwhile, red clover intercropping enhanced M% and grain yield in oats but had no such effect in barley. In both spring barley and oat, M% was positively correlated with grain yield.

## 1. Introduction

The land used for agricultural activities is a special natural resource without which humanity could not exist and feed itself. Climate change impacts are stressing land productivity and increasingly hindering efforts to meet the demands of human nutrition [1]; therefore, the conservation of land best suited for agricultural production is a global problem today.

Mixed cropping is a widely adopted sustainable agricultural practice that promotes more efficient use of resources [2,3]. Among mixed cropping systems, cereals/legumes intercropping is particularly beneficial, as it improves soil conservation [4,5], has a positive effect on soil fertility [6] and stability [7,8], contributes to long-term N immobilization [9], and benefits the companion/subsequent crops [10]. The N_2_-fixing plant can fertilize the soil directly and neighboring plants indirectly through above and belowground litter inputs, leachates from leaves, and exudates from roots. When legumes are intercropped with non-legumes, they often exhibit increased growth and yield. Such combinations are generally more productive than intercrops involving either two legume species or two non-legume species.

Moreover, the integration of legumes has been reported to have an impact on soil biological activities, especially by altering microbial community composition. This includes significant effects on soil biological activities such as those related to arbuscular mycorrhizae (AM) [11,12,13]. It is known that AM fungi are an important component of most agroecosystems and form a mutualistic symbiosis with host plant roots [14,15]. Through this association, plants can access a greater volume of soil, thereby improving the uptake of water and nutrients, enhancing the resistance to soil pathogens and drought, and improving water use efficiency [16,17] compared to plants lacking this symbiosis. Further, as pathways of root carbon to the soil [18] and agents of root carbon stabilization in the soil matrix [19]. AM fungi contribute significantly to plant growth by enhancing shoot and root biomass, which is directly involved in increasing crop yields [20,21,22].

Plant roots play a central role in the functioning of natural and agricultural ecosystems by driving the sustainable acquisition of soil resources [14,23]. Moreover, roots serve as the primary precursor of soil organic carbon (SOC), with significantly greater efficiency in contributing to SOC accumulation compared to other biomass left in the soil [24,25,26]. Crops with optimized root traits, such as deeper and more extensive root systems, can access subsoil water and nutrient reserves, making them more resilient to drought events under climate change conditions [27,28,29,30,31]. Thus, these traits are increasingly recognized as critical for future food security, contributing to both farm productivity and long-term sustainability. The root growth and development of crops depend on the plant species, soil texture, and agricultural management practices used [23,27,32,33,34,35]. Therefore, the study of root characteristics like length, diameter, volume, and symbiotic interactions is critical for understanding the functional linkages between plant performance and soil [36]. Cereal crops, i.e., oats and barley, were selected for this experiment due to their recognized importance for human health and role in dietary shifts and sustainability of agroecosystems. These crops contain relatively high levels of proteins, are rich in dietary fibers, essential vitamins and minerals, and have a naturally high disease tolerance and low nutrient requirement, and comparatively low input demand. Crop diversity has been studied within these cereal populations in combination with potential intercropping legumes—red clover and field peas. These cropping systems are currently considered to have potential for increasing vegetable protein production in Northern Europe, and they correspond to the requirement of the food industry as established through our current farming and industry network.

This experiment was designed to rigorously assess root mycorrhizal colonization intensity in the cereal/legume intercropping systems and to examine its relationship with the physical cereal root parameters and plant productivity under organic farming. This study provides new insights into the importance of mycorrhizae in agricultural practices and a knowledge gap on optimizing sustainable farming practices and more efficient fertilizer use under sustainability management practices.

## 2. Results

### 2.1. Intercropping Effect on Physical Root Parameters

**Figure 1:**
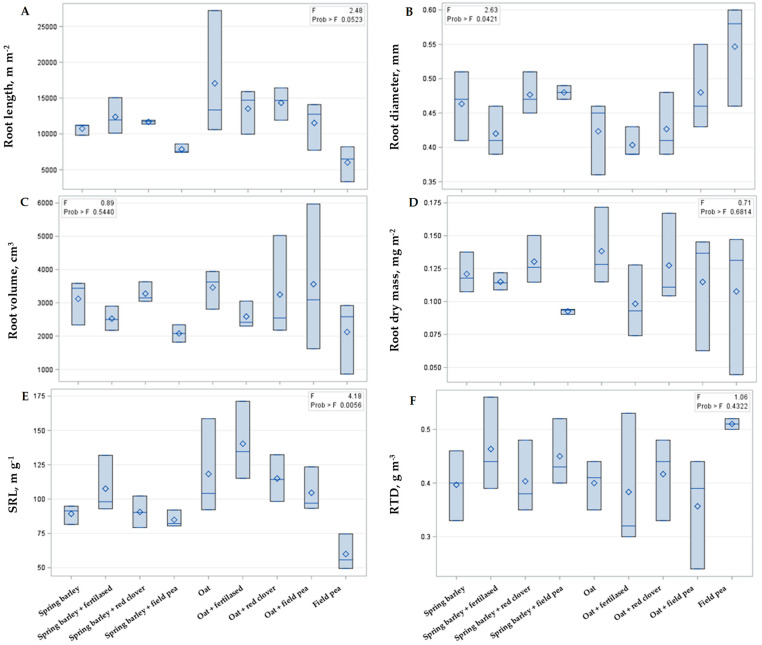
The intercropping effect on spring barley and oat root parameters. Root length (**A**), root diameter (**B**), volume (**C**), dry mass (**D**), specific root length (SRL), (**E**) and root tissue density (RTD) (**F**). In the upper right corner F refers to Fisher’s criteria, and Prob refers to probability, which is significant when *p* < 0.05, according to Duncan’s test.

The data show that three out of six root parameters had significant differences among the investigated treatments (Figure 1). The changes in root length, root diameter, and SRL indicate that intercropping caused a significant impact on crop root architecture. Field pea exhibited significantly shorter and thicker roots compared with oat and barley, which in turn affected the mean root length and diameter in the oat–pea and barley–oat cropping systems. Fertilization with granulated chicken manure also tended to increase those parameters of oat and barley monocultures, but the most significant effect was observed when red clover was intercropped with either barley or oat.

Neither dry biomass of roots (which ranged from 9.25 to 13.83 mg m^−2^ on average), nor volume (which ranged from 990 to 6000 mm^3^) and RTD (which varied from 0.25 to 0.55 m g^−1^) was significantly affected by intercropping or fertilization with granulated chicken manure of oats and barley.

### 2.2. Intercropping Effect on Mycorrhization and Cereals Yield

**Figure 2:**
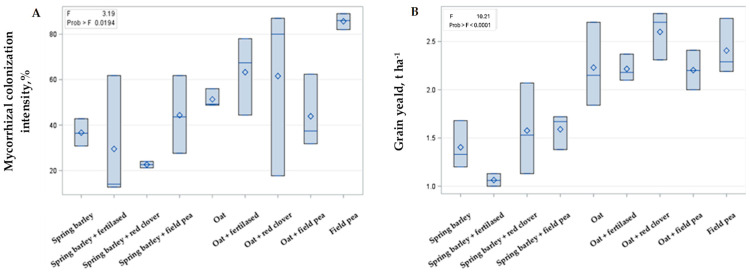
The intercropping effect on spring barley and oat mycorrhizal abundance (**A**) and grain yield (**B**). In the upper right corner F refers to Fisher’s criteria, and Prob refers to probability, which is significant when *p* < 0.05, according to Duncan’s test.

The study showed that mycorrhizal colonization and grain yields differ significantly among the investigated treatments (Figure 2). Field pea exhibited the highest mycorrhizal colonization compared with oat and barley. However, intercropping with pea did not significantly affect mycorrhizal colonization in oat–pea and barley–pea systems. The results indicate that the choice of the main crop was the main factor affecting mycorrhizal colonization within the cropping systems. Across all treatments, mycorrhizal colonization ranged from 43.9 to 63.3% in oat cropping systems and from 22.6 to 44.3% in barley cropping systems.

Grain yields of oat and spring barley ranged from 2.2 to 2.6 t ha^−1^ and from 1.1 to 1.6 t ha^−1^, respectively, across all treatments. Organic fertilization with granulated chicken manure had the greatest positive effect on yield, increasing oat and barley grain production by 18% and 12%, respectively, compared with the unfertilized treatment. Intercropping with a field pea also has a positive effect on the yields of spring barley and oat. In contrast, red clover intercropping increased oat grain yield but reduced that of spring barley.

### 2.3. Interaction Effects of Selected Species and Management Practices

**Figure 3:**
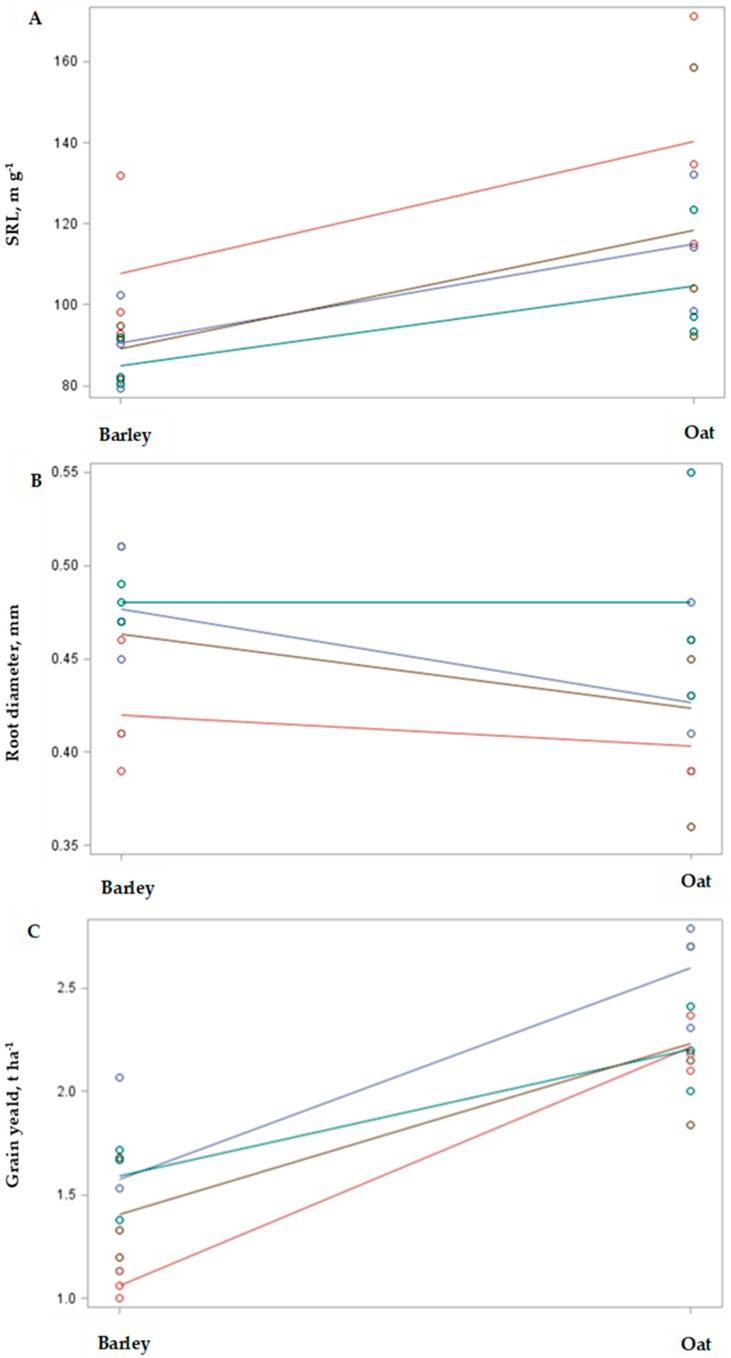

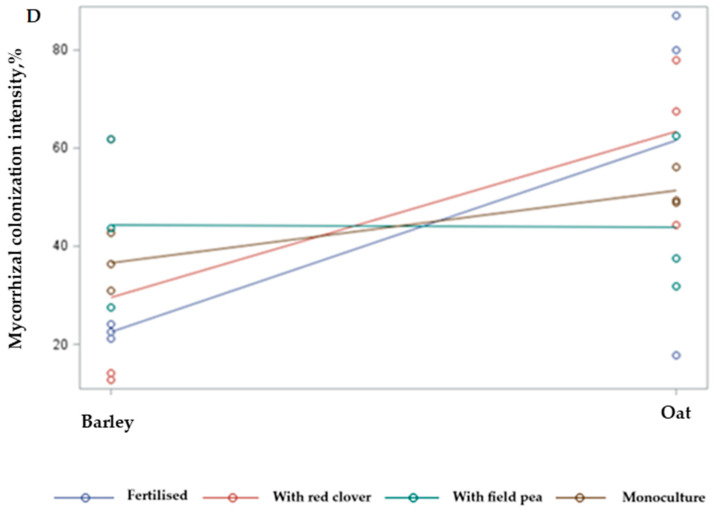
Interaction plots for specific root length (**A**), root diameter (**B**), grain yield (**C**), and mycorrhizal abundance (**D**) of oats and barley (factor 1) under different management practices: monoculture, intercropping, or fertilization (factor 2).

Interaction plots were developed to evaluate two factor groups: crop species (oat and barley intercropped with pea or red clover) and management practice (monoculture, intercropping, or fertilization instead) (Figure 3). These plots were generated for four parameters that were significantly affected by the tested factors. The results show that the choice of the main crop (oat or barley) had a significant effect on crop performance, including grain yield, mycorrhizal colonization, root diameter, and SRL.

Grain yields, mycorrhizal colonization, and root diameter were generally higher in oat compared with barley under the same management practices. Meanwhile, root diameter has similar variability for both oat and barley, but the management factors had different impacts on their performance.

Results of grain yield show that different management practices could be suggested for oats and barley. Barley benefited most from intercropping, whereas oat responded more strongly to organic fertilization with granulated chicken manure. Field pea intercropping had some positive effects on the yields of both oat and barley, while red clover intercropping did not improve oat yield and had a negative effect on barley yield.

The diameter of oat and barley roots increased with fertilization with granulated chicken manure and was further enhanced by peas in oat–pea intercropping. SRL increased in both cereals when intercropped with red clover.

Most reverse interaction effects were observed for the mycorrhizal colonization. In oat fertilization with granulated chicken manure and red clover intercropping significantly increased colonization, while in barley these practices reduced colonization. In contrast, field pea intercropping increased mycorrhizal colonization in barley but not in oat.

### 2.4. Relationship Between Physical Root Parameters, Mycorrhization Colonization, and Cereals Yield

The relationships between physical root parameters, mycorrhization colonization, and cereals yield in different intercropping systems are presented in Table 1. The experimental data showed that the root length positively correlated with root volume (r = 0.5834, *p* < 0.05), root dry mass (r = 0.6325, *p* < 0.05), and SRL (r = 0.6830, *p* < 0.05), while the root length (r = −0.5014, *p* < 0.05) and SRL (r = −0.7752, *p* < 0.01) correlation with root diameter was negative (Table 1). Mycorrhizal colonization intensity (M%) had a positive correlation with spring barley and oat yield (r = 0.6273, *p* < 0.05).

## 3. Discussion

### 3.1. Intercropping Effect on the Physical Root Parameters of the Main Crop

Intercropping is a management practice that relies on plant–plant interactions to maximize the growth and productivity within crop rotation. Studies have shown that intercropping facilitates the development of different root types, altering the overall distribution and structure of roots [37]. Roots play key roles in nutrient and water uptake, anchorage, mechanical stability, metabolite storage [38], and interactions with microorganisms [39]. Therefore, promoting interactions between different species of plant roots is particularly important for both increasing crop yield and optimizing agricultural land use efficiency [40,41,42,43]. Physical root parameters are commonly described, such as root length, volume, and diameter [44]. This study indicates that intercropping significantly affected these parameters in the main crops. Field pea had significantly shorter and wider roots compared with oat or barley, thereby impacting the general mean root length and diameter in oat–pea and barley–pea cropping systems. Fertilization with granulated chicken manure also tended to increase those parameters of oat and barley monocultures. However, the strongest effects were observed when red clover was intercropped with either barley or oat. Root diameter in both cereals increased under fertilization with granulated chicken manure and was further enhanced when pea was intercropped with oat.

In ecological studies, the functions of plant species in ecosystems are often evaluated through ‘integrated’ traits that are measured at the plant level or even at the community level, such as SRL and RTD. SRL are plant parameters that show the interspecific belowground interactions [45]. Plants with high SRL build more root length for a given dry mass investment and are generally considered to have higher rates of nutrient and water uptake (per dry mass), shorter root lifespan than low SRL plants. This study shows increased SRL in both oat and barley intercropped with red clover, and elevated SRL values were also observed when intercropped with pea. This suggests that intercropping with legumes can promote root system adjustments that enhance soil resource acquisition efficiency. RTD is considered an important predictor of plant strategies [46,47] since it is commonly associated with many critical aspects of plant growth and survival. Low-density tissues enable a fast relative growth rate and a rapid resource acquisition, as the plant can rapidly expand its root system with a low investment in dry matter [46,48,49]. However, our results did not show that RTD was significantly affected by intercropping or organic fertilization with granulated chicken manure in oat and barley. Therefore, our study gives practical guidance to better adapt to climate change and build resilient cropping systems through intercropping.

### 3.2. Intercropping Effect on the Mycorrhization Intensity and Its Interaction with Main Crop Yield

The effect of cereal–legume intercropping on crop yield has been extensively researched and documented across agricultural studies. This agricultural practice effectively enhances nitrogen levels in the soil, thereby directly influencing the grain yield. These investigations highlight a notable increase in the cereal yield when intercropped with legumes, compared to being grown using monoculture practices. Most of the previous intercropping studies have reported a number of benefits linked to the integration of legumes into cereal cropping systems, i.e., yield stability, enhanced resource use, and harvesting efficiency [50,51,52,53]. In our study, the yield response of cereals varies depending the on species and management practice. Field pea intercropping improved the yields of both cereals, while red clover intercropping did not impact yield, and negatively affected the grain yield of barley. Intercropping had a more positive effect on barley, whereas oats responded more strongly to organic fertilization. According to this tendency, agricultural practices should be revised when it comes to practice. While oats are more commonly cultivated as mixtures than barley, barley systems are more fertilized than oats.

A few intercropping studies have examined mycorrhizal colonization in different intercropping systems and their effect on yield [20,21,22,54]. In agricultural settings, the arbuscular mycorrhizal fungi (AMF) community composition and diversity can change over time depending on the crop species and management practice in place, highlighting the need to adopt cropping systems that stimulate the proliferation of AMF communities important in providing essential soil ecosystem services [12,13,54,55]. Red clover and field pea are known to form strong mycorrhizal associations, with reported colonization rates ranging from 6 to 46% in red clover [56,57] and 16–67% in field pea [58,59]. These colonization levels indicate that cover crops and intercrops could have significantly influenced the AMF community structure or abundance in the rhizosphere, potentially affecting the colonization observed in the main crops. However, studies on changes in the diversity and composition of the mycorrhizal community induced by various intercrop species have yielded variable results [12,13,20,21,22,56,57,58,59]. Zhang et al. [60] showed that intercropping promoted diversity and shaped the structure of AMF communities in soil and root compartments, compared to the respective main crops. Lu [54] and Bini et al. [61], respectively, showed that intercropping increases AMF activity and diversity in the soil, as a result of modified soil biochemical properties. Koskey et al. [62] who also found that intercropping enhanced AMF abundance, activity, and colonization compared to those of the main crops. Our results revealed contrasting interaction effects of management practices on mycorrhizal colonization in oat and barley. Fertilization with granulated chicken manure and intercropping with red clover significantly increased mycorrhization in oat, while these practices reduced colonization in barley. In contrast, field pea increased the mycorrhizal colonization in barley but not in oat. These results confirm the observations of the productivity of crops and suggest revising agricultural practices also based on evidence of ecosystem services such as mycorrhization. Therefore, future studies in similar cropping systems should include AMF assessments for all co-occurring plant species to better understand system-level mycorrhizal dynamics and their role in shaping treatment effects. In the Nemoral zone of Europe, oats are more commonly cultivated as mixtures than barley, but our study suggests a higher need of barley being intercropped with plants such as field pea. Contrastingly, barley systems are more frequently fertilized than oats in practice, but our study shows higher advantages of oat being slightly fertilized with organic manure or sown with red clover intercrop, than using standard diversification practice of oat intercropped with field pea.

Besides the influence of crop species, AMF community diversity and composition changes due to crop cultivars have been detected. For instance, different wheat cultivars may select specific AMF phylotypes colonizing the roots, signifying a complex pattern of plant–AMF interactions even at the genotype level [12,63]. Thus, the choice of the intercrop partner in relay intercropping could be critical. In general, arbuscular mycorrhizal fungi (AMF) play an important role in intercropping systems in two main ways: protecting plant health and improving plant growth and yield. Studies on yield response to naturally occurring mycorrhizal abundance of different crop systems are comparably scarce [64,65]. Boudabbous et al. [64] found the positive effect of mycorrhizal abundance on grain yield, and Kirk et al. [66] found that no significant correlation was observed between yield and AMF. In our study, we observed that mycorrhizal colonization intensity (M%) showed a positive and significant correlation with spring barley and oat yield (r = 0.6273, *p* < 0.05). Hence, higher mycorrhization intensity generally leads to increased yields, but increasing crop performance may not be the only benefit AMF can provide [67]. Moreover, genotypic differences in, e.g., morphological properties of the root system or crop physiology may obscure benefits of mycorrhization for yield, which need to be studied in more detail in field experiments [65].

## 4. Materials and Methods

### 4.1. Experimental Site

**Figure 4:**
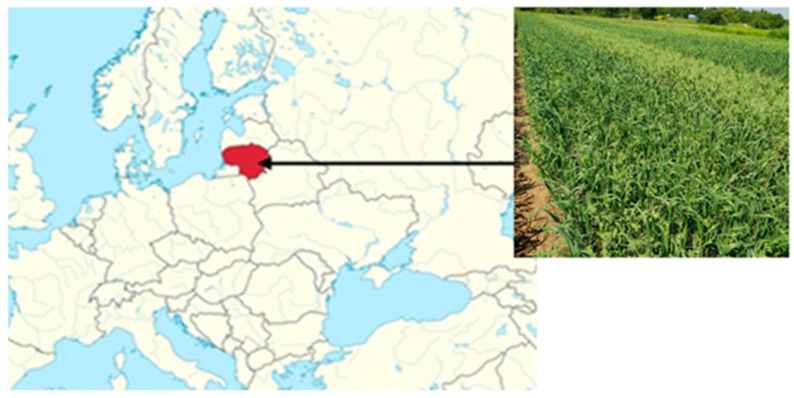
Study site location.

The study was carried out at the Lithuanian Research Centre for Agriculture and Forestry (LAMMC), Institute of Agriculture in Central Lithuania’s lowland regions, 55°24′ N, 23°51′ E (Figure 4) in 2024. The field experiment was conducted in the organically managed cropping system with a sandy loam texture soil in which the content of clay particles (<0.002 mm) was 14.8%, silt (0.063–0.002 mm) 36.7% and sand (2.0–0.063 mm) 48.5%, bulk density 1.8 Mg m^−3^. Experimental soil type was classified as *Endocalcary-Endohypogleyic Cambisol* according to WRB.

The chemical characteristics on the top-soil layer (0–30 cm) in spring, before establishing the experiment, were as follows: pH of 7.5, 74–79 mg kg^−1^ available phosphorus (P_2_O_5_), 135–140 mg kg^−1^ potassium (K_2_O) 7.12 mg kg^−1^ DM of nitrate-nitrogen (NO_3_-N), 4.36 mg kg^−1^ DM of ammonium-nitrogen (NH_4_-N), and a high humus content—2.3%.

The site has been managed organically since 2003, without mineral fertilization, herbicides, pesticides, or irrigation. The farming type is exceptionally focused on crop production, where N is supplied by a diversity of grain and forage legume plants, plant-based fertilizers, and microbial substances.

### 4.2. Meteorological Conditions

The climate in Lithuania is classified as humid continental, with warm summers and relatively severe winters. According to standard climate norm data (1924–2024), the average air temperature at the experimental site was 9.6 °C, and the average amount of precipitation amounted to 586.2 mm. The crop vegetation period in 2024 was warmer and drier than usual, which created unfavorable conditions for plant growth (Table 2). In 2024, the average air temperature during the plant’s vegetation period was 2.4 °C higher and the total amount of precipitation was 63.3 mm lower compared to the long-term mean.

### 4.3. Experimental Design and Treatments

The field experiment was conducted from April to August 2023 as part of an established crop rotation system consisting of common barley (*Hordeum vulgare* L.) → red clover (*Trifolium pratense* L.) → winter wheat (*Triticum aestivum* L.) → spring crop mixtures. The field was plowed in the autumn and harrowed in the spring. The experiment was based on a randomized complete block design with four replicates. Each plot was 1.5 m in width and 5.0 m in length, with a standard row spacing of 15 cm. Three experimental factors were tested: agronomic practice (mixtures vs. monocultures), crop species (spring barley, oat, field pea, and red clover), and fertilization (fertilized with organic granulated chicken manure and no fertilization). The experiment consisted of the following treatments: 1—spring barley, 2—spring barley fertilized with organic granulated chicken manure, 3—spring barley with red clover, 4—spring barley with field pea, 5—oat, 6—oat fertilized with organic granulated chicken manure, 7—oat with red clover, 8—oat with field pea, and 9—field pea. Spring barley and oats were the main crops compared with each other. Field pea and red clover were used as intercrops for the main crop. Field pea acted as a supplement to the main crop for the yield in the equal ratios, while red clover acted as a cover crop, not for the seeds, but for the biomass outcome. The varieties and rates of sowing were as follows: oat Viva DS 3.0 million seeds ha^−1^, spring barley Noja DS 3.0 million seeds ha^−1^, field pea—Jura DS 3.0 million seeds ha^−1^, and red clover—Arimaiciai DS 3.0 million seeds ha^−1^. In the mixtures of oats and barley with field pea, a 0.5:0.5 ratio of full rate were used for both crops. In the mixtures of oats and spring barley with red clover, full rates of both mentioned before was used. Seeds of the mixtures were sown together at the same time to the same rows of 12.5 cm. All crops were sown on 27th April and harvested on 24th August. One treatment for oat and barley was fertilized with granulated chicken manure and consisted of 5% N, 4% P, and 2% K. The quantity was calculated to deliver 30 kg N per hectare and applied in the spring before the sowing the main crop.

### 4.4. Mycorrhizal Colonization Intensity (M%)

**Figure 5:**
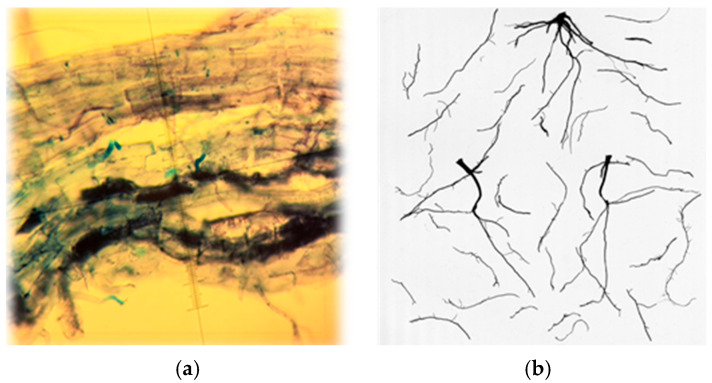
Investigation of the root system: (**a**) measurement of mycorrhizal colonization intensity in the root segments; (**b**) measurement of root architecture parameters.

Root samples for assessing mycorrhizal colonization intensity (M%) in spring barley and oat were collected at the flowering stage (BBCH 63–65). Samples were taken from a 0–15 cm soil depth, with roots from 10 individual plants per plot pooled into a single composite sample. The samples were immediately transported to the lab at ambient temperature, where the roots were carefully separated from the soil during a washing process. They were then cut into approximately 1.5 cm segments, stained with 0.05% (*w*/*v*) methyl blue in lacto-glycerol (1:1:1 lactic acid, glycerol, and water) for 1 min, and destained with distilled water for 1 min more [68]. The stained root fragments were placed on prepared slides, mounted in glycerol, and examined under a light microscope (Nikon, Nikon Eclipse E100, Tokyo, Japan) at the LAMMC Microbiology Laboratory (Figure 5a). The mycorrhizal colonization intensity (M%) of roots was quantified following the method of Trouvelot, Kouch, and Gianinazzi-Pearson [69], which is based on the observation of the root fragments occupied by AMF structures. For each plant and treatment, the total number of observed fragments was three plants × three blocks × 10 = 90. M% was calculated by attributing to each root fragment increasing scores from 0 to 5: 0 = no AMF structures within the root segment; 1 = structures occupy <1% of the root segment; 2 = structures occupy <10% of the root segment; 3 = structures occupy <50% of the root segment; 4 = structures occupy more than 50% of the root segment; and 5 = structures occupy more than 90% of the root segment [70].

### 4.5. Investigations of Physical Root Parameters

The root development of spring barley and oat crop was assessed once during the growing season at the flowering stage (BBCH 61–65). Samples were collected using the small monolith method (10 × 10 × 10 cm) with three replications per treatment plot. The collected samples were tightly packed into plastic bags and stored in a freezer at −20 °C until analyzed. Prior to analysis, soil and root samples were carefully washed with water using 500 and 250 µm sieves. The cleaned root samples were stained with methylene blue solution (0.5% (*w*/*v*)), spread separately in a thin layer of water (1–3 mm) in a glass tray, and arranged with mounted needles. Root systems were scanned with a flatbed scanner (EPSON perfection V700; Seiko Epson Corp., Suwa, Japan) at a resolution of 800 dots per inch in transparency mode (Figure 5b). For each species, 6 to 10 images were recorded and analyzed using WinRhizoPRO software (V2012b, Regent Instruments, Québec, QC, Canada) to determine root length (m m^−2^), average root diameter (mm), and root volume (cm^3^). After scanning, root samples were oven-dried for 48 h at 60 °C and weighed to determine their dry mass (mg m^−2^). Specific root length (SRL, in m g^−1^) was calculated as the ratio of root length to dry mass (m g^−1^), while root tissue density (RTD, in g cm^−3^) was calculated as the ratio of dry mass to root volume [71].

### 4.6. Crop Productivity

The productivity of spring barley, oat, and field pea was determined as grain yield after harvesting at the standard moisture (14%).

### 4.7. Statistical Analysis

The data of mycorrhizal abundance, physical cereal root parameters and crop productivity were statistically processed using the software program SAS Enterprise Guide 7.1 by multivariate analysis of variance (ANOVA). One-way Anova was performed for the 9 treatments listed in Section 4.3. Then, two-way Anova was performed using factors: agronomic practice (mixtures, monocultures, or fertilization) and crop species used as main crops (spring barley or oat). The interaction plots were generated for those treatments. The Duncan’s multiple range tests were applied to compare the mean values and significant differences between means at the level of *p* < 0.05. Analysis of the relationship between different parameters was performed using Pearson’s correlation.

## 5. Conclusions

This study demonstrated that intercropping with field pea and red clover can have a positive effect on main crop root architecture parameters, mycorrhizal colonization, which showed a strong relation with grain yield in an organic system. The most significant effect on spring barley and oat root architecture parameters was in intercropping with red clover. The effects on mycorrhizal colonization (M%) and yield, however, differed between crops: field pea intercropping significantly increased M% in spring barley and enhanced its grain yield, whereas red clover intercropping improved both M% and yield in oat. Overall, these findings highlight the potential of cereal/legume intercropping to optimize root traits, improve resource acquisition, and enhance crop productivity under organic farming conditions. The results suggest that cereal/legume intercropping is a promising tool for sustainable agriculture in order to maintain yield performance.

## Figures and Tables

**Table 1:** Physical root parameters, mycorrhization, and cereals yield correlation matrix.

Correlation Matrix								
	Root Length, m m^−2^	Root Diameter, mm	Root Volume, cm^3^	Root Dry Mass, mg m^−2^	SRL, m g^−1^	RTD, g cm^−3^	M, %	Cereals Yield, t ha^−1^
**Root length, m m^−2^**	1.0000	−0.5014 *	0.5834 *	0.6325 *	0.6830 *	−0.2758	−0.1739	0.0806
**Root diameter, mm**	−0.5014 *	1.0000	0.2676	0.1858	−0.7752 **	−0.1124	0.1747	0.0917
**Root volume, mm^3^**	0.5834 *	0.2676	1.0000	0.7596 **	0.0989	−0.6507 *	−0.1747	0.0954
**Dry mass, mg m^−2^**	0.6325 *	0.1858	0.7596 **	1.0000	−0.0915	−0.0694	−0.1847	0.0270
**SRL, m g^−1^**	0.6830 *	−0.7752 **	0.0989	−0.0915	1.0000	−0.3892	−0.0242	0.1195
**RTD, g cm^−3^**	−0.2758	−0.1124	−0.6507 *	−0.0694	−0.3892	1.0000	0.0713	−0.1222
**M, %**	−0.1739	0.1747	−0.1747	−0.1847	−0.0242	0.0713	1.0000	0.6273 *
**Cereals yield, t ha^−1^**	0.0806	0.0917	0.0954	0.0270	0.1195	−0.1222	0.6273 *	1.0000

*—significant when *p* < 0.05; **—significant when *p* < 0.01.

**Table 2:** Meteorological conditions: annual mean air temperature (°C) and total amount of precipitation (mm) during the study period, 2024. The data is from the Dotnuva Meteorological Station.

Year	2024	Long—Term Mean (1924–2024)
Annual mean air temperature, °C	9.6	6.6
Difference from long—term mean, °C	+3.0	−
Average air temperature during the plant’s vegetation period, °C	18.2	15.8
Difference from long—term mean, °C	+2.4	−
Total annual precipitation, mm	586.2	569.3
Difference from long—term mean, mm	+16.9	−
Total amount of precipitation during the plant’s vegetation period, mm	200.3	263.6
Difference from long—term mean, mm	−63.3	−

## Data Availability

The original contributions presented in the study are included in the article; further inquiries can be directed to the corresponding authors.
